# How well are we engaging the family in family-based treatment for adolescent anorexia nervosa?

**DOI:** 10.1186/2050-2974-3-S1-O49

**Published:** 2015-11-23

**Authors:** Elizabeth K Hughes, Claire Burton, Daniel Le Grange, Susan M Sawyer

**Affiliations:** 1 University of Melbourne, Melbourne, Australia; 2 Royal Children's Hospital, Melbourne, Australia; 3 Murdoch Childrens Research Institute, Melbourne, Australia; 4 University of California San Francisco, San Francisco, CA, USA

## 

According to manualised family-based treatment (FBT) for adolescent anorexia nervosa, all family members should attend treatment sessions. However, there is very little research on the degree to which individual family members are involved in clinical practice. We examined the attendance rates of 198 families who participated in FBT at the Royal Children's Hospital Eating Disorders Program. Mothers' attendance was mostly stable over 6 months of FBT, ranging between 91-99% (mean=95%). Fathers' attendance declined slowly over time from a high of 83% down to 61%. (mean=71%). Siblings' attendance was 44% in the first week followed by a rapid decline to a low of 8% (mean=20%). In total, mothers attended 91% of all sessions, fathers attended 69% of sessions, and siblings attended 19% of sessions. In this presentation, we will discuss the factors that allow our service to achieve relatively high rates of paternal engagement. This includes therapeutic strategies used by clinicians to overcome resistance and service processes that enable and encourage fathers to be involved. We will also discuss factors that may affect sibling attendance and how we can improve engagement with siblings.

